# Engineering Production of a Novel Diterpene Synthase Precursor in *Nicotiana benthamiana*

**DOI:** 10.3389/fpls.2021.757186

**Published:** 2021-10-20

**Authors:** Edith C. F. Forestier, Geoffrey D. Brown, David Harvey, Tony R. Larson, Ian A. Graham

**Affiliations:** ^1^Department of Biology, Centre for Novel Agricultural Products, University of York, York, United Kingdom; ^2^Department of Chemistry, University of Reading, Reading, United Kingdom

**Keywords:** plant diterpenes, *Nicotiana benthamiana*, GGPP, hydroxy-GGPP, metabolic engineering

## Abstract

Diterpene biosynthesis commonly originates with the methylerythritol phosphate (MEP) pathway in chloroplasts, leading to the C_20_ substrate, geranylgeranyl pyrophosphate (GGPP). The previous work demonstrated that over-expression of genes responsible for the first and last steps in the MEP pathway in combination with *GERANYLGERANYL PYROPHOSPHATE SYNTHASE* (*GGPPS*) and *CASBENE SYNTHASE* (*CAS*) is optimal for increasing flux through to casbene in *Nicotiana benthamiana*. When the gene responsible for the last step in the MEP pathway, *4-HYDROXY-3-METHYLBUT-2-ENYL DIPHOSPHATE REDUCTASE* (*HDR*), is removed from this combination, casbene is still produced but at lower amounts. Here, we report the unexpected finding that this reduced gene combination also results in the production of 16-hydroxy-casbene (16-OH-casbene), consistent with the presence of 16-hydroxy-geranylgeranyl phosphate (16-OH-GGPP) in the same material. Indirect evidence suggests the latter is formed as a result of elevated levels of 4-hydroxy-3-methyl-but-2-enyl pyrophosphate (HMBPP) caused by a bottleneck at the HDR step responsible for conversion of HMBPP to dimethylallyl pyrophosphate (DMAPP). Over-expression of a *GERANYLLINALOOL SYNTHASE* from *Nicotiana attenuata (NaGLS)* produces 16-hydroxy-geranyllinalool (16-OH-geranyllinalool) when transiently expressed with the same reduced combination of MEP pathway genes in *N. benthamiana*. This work highlights the importance of pathway flux control in metabolic pathway engineering and the possibility of increasing terpene diversity through synthetic biology.

## Introduction

Many of the gem-dimethylcyclopropyl class of bioactive casbene-derived diterpenoids from plants have been shown to have pharmacological activities. Low abundance in their natural host (Hohmann et al., 2000; Johnson et al., 2008) along with their structural complexity (Jorgensen et al., 2013; Kawamura et al., 2016; Hashimoto et al., 2017) has led to efforts to engineer alternative microbial (Hill et al., 1996; Callari et al., 2018; Wong et al., 2018), algal (Mehrshahi et al., 2020), or plant-based production platforms (Forestier et al., 2021). The majority of diterpenes in plants are biosynthesized in chloroplasts (Rohmer et al., 1993; McGarvey and Croteau, 1995; Lichtenthaler et al., 1997) from the five-carbon building blocks, isopentyl pyrophosphate (IPP) and dimethylallyl pyrophosphate (DMAPP), provided by the MEP pathway and combined together in a three-to-one ratio to form the C_20_ precursor, GGPP (Gershenzon and Croteau, 1993).

We recently developed a *Nicotiana benthamiana* platform optimized for production of casbene and derivatives by engineering flux through the MEP pathway (Forestier et al., 2021). We demonstrated how this platform could be used for production of the lathyrane jolkinol C by introduction of functionally characterized P450 oxidases (Forestier et al., 2021). The elucidation of the biosynthetic steps from casbene to the tigliane (Kulkosky et al., 2001; Kissin and Szallasi, 2011; De Ridder et al., 2021), ingenane (Siller et al., 2010) and jatrophane (Corea et al., 2009; Hadi et al., 2013) classes of diterpenoids has also been investigated but remains to be resolved.

In our work on *N. benthamiana* to optimize flux through the MEP pathway to GGPP – the substrate for casbene production – we unexpectedly detected the novel compound, 16-hydroxy-casbene. The design of our experiments suggested that this metabolite did not arise from a hydroxylation downstream of GGPP and we therefore hypothesized that 16-hydroxy-casbene could derive from an alternative substrate. Herein, we present results leading us to conclude that 16-hydroxy-GGPP can act as a novel precursor for diterpene biosynthesis.

## Materials and Methods

### Transient Expression of Genes in *Nicotiana benthamiana*

*Arabidopsis thaliana* cDNAs of *1-DEOXY-D-XYLULOSE-5-PHOSPHATE SYNTHASE* (*DXS*, AT4G15560.1), *4-HYDROXY-3-METHYLBUT-2-ENYL DIPHOSPHATE REDUCTASE* (*HDR*, AT4G34350.1, Phillips et al., 2008), and *GERANYLGERANYL PYROPHOSPHATE SYNTHASE 11* (*GGPPS11*, AT4G36810.1, Beck et al., 2013) coding for plastidial enzymes referenced in TAIR (The Arabidopsis Information Resource) and cDNA of *CASBENE SYNTHASE* (*CAS*) from *Jatropha curcas* (King et al., 2014) were cloned into pEAQ-HT (Sainsbury et al., 2009) vector as described in Forestier et al. (2021). A codon optimized cDNA of *NaGLS* based on the sequence accession number XM_019410085 was synthesized by gBlock IDT with extensions allowing to clone it directly with In-Fusion® in AgeI/StuI linearized pEAQ-HT vector. Transient expression in wild-type *N. benthamiana* was performed as described in Forestier et al. (2021).

### Isolation and Quantification of Diterpenoids, GGPP, and OH-GGPP

We detected both casbene and 16-OH-casbene in transiently expressed plants by extracting around 200mg of dry material with 5ml of hexane containing 100μg/ml of β-caryophyllene, then sonicating for 15min. We quantified the compounds by GC–MS as detailed in Forestier et al. (2021). For geranyllinalool and its derivatives, we extracted around 150mg of dry weigh (DW) of infiltrated tobacco with 1ml of ethyl acetate containing 100mg/L of β-caryophyllene. The samples were shaken overnight at 2000rpm on a IKA Vibrax VXR basic shaker and then centrifuged, and 100μl of the supernatant was used directly for GC–MS.

We isolated GGPP and 16-OH-GGPP by adapting the protocol described by Nagel et al. (2014). Approximately 750mg of ground dry material was extracted with 15ml of methanol/H_2_O (7:3, v/v) and sonicated for 30min. We then added 5ml of water to the mixture, centrifuged for 3min at 2000*g*, and filtered through Whatman filter paper grade 1 and cotton. The cleared extracts were passed through Chromabond HX RA columns and pre-conditioned with 5ml of methanol and 5ml of water, and compounds were eluted with 3ml of ammonium formate 1M in methanol. Each eluate was dried under a stream of nitrogen and re-dissolved in 250 μl of water/methanol (1:1). We transferred 100 μl into glass HPLC vials, and 2 μl aliquots were analyzed by LC–MS as described by Catania et al. (2018). Additional high-resolution mass spectral data were obtained on a parallel LC interfaced to a Thermo Orbitrap Fusion mass spectrometer, operating in ESI mode at 500000 (FWHM) resolution for MS1 data, with MS2 data collected at 120000 resolution using stepped collision energies between 20 and 60units in both HCD and CID modes.

### Accumulation and Purification of Compounds for NMR Spectroscopy

To identify 16-OH-casbene, we ground 4.9g of dry material obtained from 10 full-grown plants infiltrated with *DXS*, *GGPPS*, and *CAS*, and extracted with 100ml of hexane. After 1h of sonication and 2days shaking, the extract was centrifuged for 3min at 2000*g*, filtered through Whatman paper grade 1 and cotton, and evaporated to obtain 350mg of oily residue. The residue was re-suspended in 10ml of hexane/ethyl acetate (70:30, v/v) and purified through a 40g Buchi silica column on a PuriFlash® 4,250 system (Interchim). We used the same method of flash chromatography as described in King et al. (2014) to fractionate the extract into 80 samples. GC–MS was used to identify the fraction containing our compound of interest, and 2.6mg of this was obtained after evaporation, at sufficient purity for direct ^1^H NMR analysis on a Bruker AVIII 700MHz instrument equipped with a cryoprobe.

For 16-OH-geranyllinalool, we infiltrated 40 young plants with *DXS*, *GGPPS*, and *NaGLS*, which provided 10g of dry material after freeze-drying and grinding. We extracted this with 150ml of ethyl acetate and left to shake for 5days on a rotary shaker. After centrifugation and filtration as detailed above, we reduced the volume down to 1ml before re-suspending in 9ml of hexane/ethyl acetate and purifying with the same column and method as described above. The fractions of interest were combined and dried to obtain 16.4mg of extract that was further purified with a reverse phase column [C18-HQ 5μm 250 mm×10 mm (Interchim)] to remove the pigment content. The reverse phase column was first equilibrated with solvent A – mix of water/acetonitrile (95:5, v/v) – for eight column volumes (CV), before injecting the extract, diluted in 2.5ml of the same solvent, into a 5ml injection loop. The separation method consisted of one CV of solvent A, followed by a gradient of nine CV, to reach 100% acetonitrile (solvent 0B). This solvent was maintained for a further 10 CV, and the entire run was carried out at a flow rate of 3ml/min. We used an in-line connected Advion Expression compact mass spectrometer (CMS), which enabled product isolation guided by mass spectra. To evaluate the fragmentation of 16-OH-geranyllinalool, we additionally ran the extracts on UPLC-MS, allowing us to determine two main ions at m/z 271 and m/z 289, which we used to select our compound of interest on the Puriflash-CMS. We collected one fraction, which after evaporation contained 4.6mg of sufficiently pure metabolite for NMR identification.

## Results

### Transient Expression of *1-DEOXY-D-XYLULOSE-5-PHOSPATE SYNTHASE* With *GERANYLGERANYL PYROPHOSPHATE SYNTHASE* and *CASBENE SYNTHASE* in *Nicotiana benthamiana* Can Produce Metabolites in Addition to Casbene

**Figure 1:**
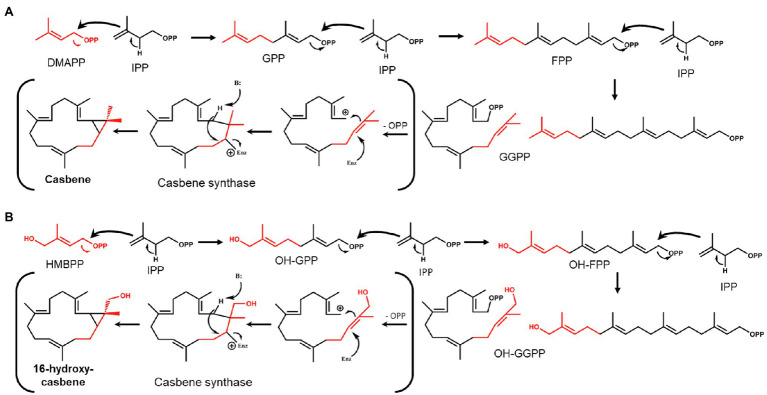
GC–MS total ion chromatograms (TIC) showing the diterpene profile of *N. benthamiana* infiltrated with casbene synthase (CAS) and different precursor genes. **(A)** Co-expression of *DXS*+*GGPPS*+*CAS*. **(B)** Co-expression of *DXS*+*HDR*+*GGPPS*+*CAS*. **(C)** Simplified diagram of the MEP pathway plus geranylgeranyl pyrophosphate synthase (GGPPS). MEP pathway enzymes are DXS (1-deoxy-D-xylulose 5-phosphate synthase), DXR (1-deoxy-D-xylulose 5-phosphate reductoisomerase), MCT (2-C-methyl-D-erythritol 4-phosphate cytidylyltransferase), CMK (4-(cytidine 5’diphospho)-2-C-methyl-D-erythritol kinase), MDS (2-C-methyl-D-erythritol 2,4-cyclodiphosphate synthase), HDS (4-hydroxy-3-methylbut-2-enyl-diphosphate synthase), HDR (4-hydroxy-3-methylbut-2-enyl diphosphate reductase), and IPPI (isopentenyl diphosphate Δ-isomerase). 1, casbene; 2, 16-hydroxy-casbene; and 3 and 4, undetermined compounds.

In the previous work, we tested the transient co-expression of different MEP pathway genes and *GGPPS* from *A. thaliana* with *CAS* from *Jatropha curcas* to evaluate the best combination for the highest production of casbene. We determined that the combination of *DXS* (catalyzing the first step in the MEP pathway), *HDR* (4-hydroxy-3-methylbut-2-enyl diphosphate reductase, catalyzing the last step), *GGPPS*, and *CAS* resulted in an up to 5-fold increase in casbene production, compared to *CAS* expression alone (Forestier et al., 2021). Omitting *HDR* from this combination resulted in lower production of casbene (Forestier et al., 2021). Further inspection of the total ion chromatograms of plant extracts from this reduced gene combination identified three additional peaks (Figure 1A), compared to co-expression of *DXS*, *HDR*, *GGPPS*, and *CAS* which only produced casbene (Figure 1B).

The largest of the three additional peaks was present in sufficient amount to allow its identification as 16-hydroxy-casbene (16-OH-casbene) by NMR spectroscopy (Supplementary Figure S1). 16-OH-casbene was present at approximately 30% of casbene levels when *HDR* was absent from the gene combination (Supplementary Figure S2).

**Figure 2:**
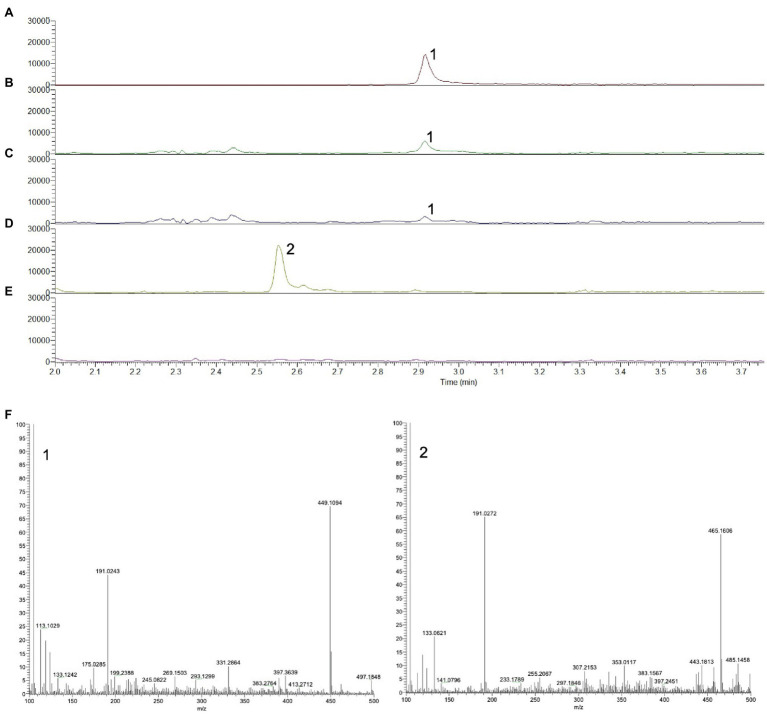
Theoretical formation of GGPP/casbene and OH-GGPP/16-OH-casbene. **(A)** Condensation of dimethylallyl pyrophosphate (DMAPP) and isopentyl pyrophosphate (IPP) to form GGPP and cyclisation by casbene synthase to form casbene. **(B)** Condensation of HMBPP and IPP to form OH-GGPP and cyclisation by casbene synthase to form 16-OH-casbene.

The fact that 16-OH-casbene is only produced when *HDR* is omitted from the gene combination used to increase flux into the C_20_ GGPP precursor for casbene production, led us to investigate the possibility that the hydroxyl group at the 16-position of the precursor is also hydroxylated. GGPP is formed by head-to-tail condensations between one molecule of DMAPP and three molecules of IPP (Ogura and Koyama, 1998), with the chain-starter DMAPP ending up distal to the pyrophosphate group (Figure 2A). In the MEP pathway, the immediate precursor to DMAPP is (*E*)-4-hydroxy-3-methyl-but-2-enyl pyrophosphate (HMBPP; Figure 1C), which has the same structure as DMAPP, but with a hydroxyl functionality at position 4 (Figures 2A,B). This hydroxyl group would appear at the 16-position of GGPP after chain-extension by IPP if HMBPP is accepted by GGPPS as an alternative chain starter to DMAPP (Figure 2B). We considered therefore that 16-OH-GGPP could be formed if there were an excess of HMBPP, caused by the over-expression of *DXS* and *GGPPS* with insufficient conversion to DMAPP and/or IPP due to this being dependent on an endogenous *HDR*. 16-OH-GGPP could then be further incorporated into the casbene backbone assuming that *CAS* accepts 16-OH-GGPP as substrate for production of 16-OH-casbene (Figure 2B).

### Detection of GGPP and Putative 16-OH-GGPP *in Planta* by Transient Over-Expression of *DXS*+*GGPPS* and *DXS*+*HDR*+*GGPPS*

**Figure 3:**
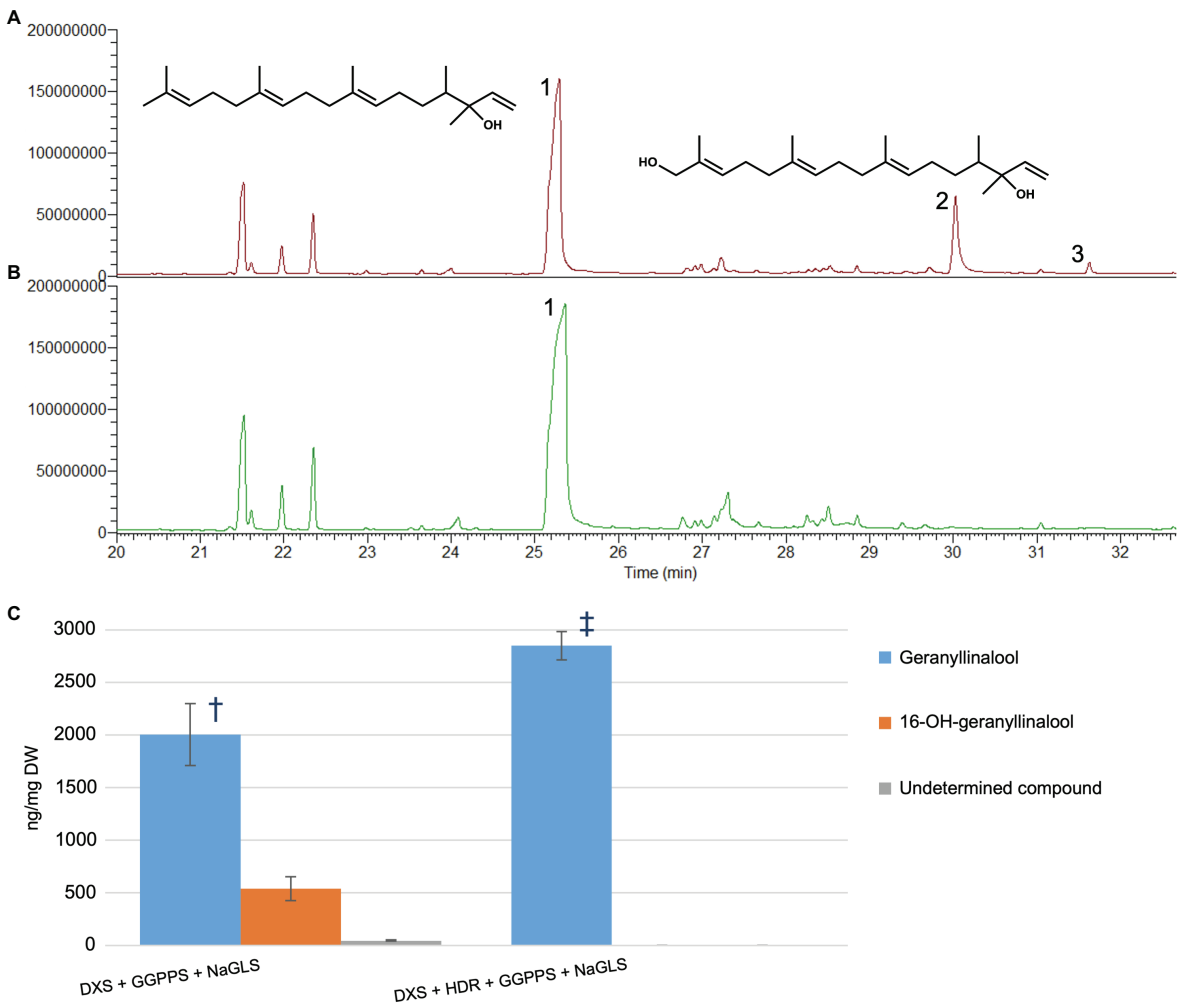
UPLC-MS chromatograms showing the prenyl diphosphate profile of *N. benthamiana* infiltrated with different diterpene precursor genes. **(A)** Authentic standard of GGPP, m/z 449–450. **(B)** Co-expression of *DXS*+GGPPS, m/z 449–450. **(C)** Co-expression of *DXS*+*HDR*+*GGPPS*, m/z 449–450. **(D)** Co-expression of *DXS*+*GGPPS*, m/z 465–466. **(E)** Co-expression of *DXS*+*HDR*+*GGPPS*, m/z 465–466. **(F)** Mass spectrum of GGPP (1) and putative OH-GGPP (2).

To establish whether 16-OH-GGPP accumulates depending on the gene combination, either *DXS*+*GGPPS* or *DXS*+*HDR*+*GGPPS* were transiently expressed in *N. benthamiana* and C_20_ prenyl diphosphate intermediates were extracted as described by Nagel et al. (2014). UPLC-MS/MS negative mode analysis of methanolic extracts was used to detect GGPP by selecting the m/z range 449–450 and by comparison with an authentic GGPP standard (Figures 3A–C). In the absence of a 16-OH-GGPP standard, we predicted that since m/z 449.2 represents the [M-H]- ion for GGPP (Figure 3F), hydroxylated forms should be detectable at an added mass of 16; i.e at 465.2. Hydroxylated forms of prenyl diphosphates would be more hydrophilic and therefore elute at an earlier retention time compared to GGPP in reverse phase chromatographic separation. A clear peak at m/z 465.2, was detected at the earlier retention time of 2.5 min, in the extract of material over-expressing *DXS*+*GGPPS* (Figures 3D, F), consistent with 16-OH-GGPP. High-resolution mass spectrometry analysis revealed a m/z of 449.1869 for GGPP ([M-H]^−^ theoretical 449.1864; error 1.11ppm) and m/z of 465.1819 for 16-OH-GGPP ([M-H]^−^ theoretical 465.1813; error 1.29ppm). Both peaks generated a common 158.9252m/z MS2 fragment, identified as the diagnostic pyrophosphate group ion, [P_2_O_6_H]^−^. We were unable to detect the m/z 465.2 peak in the gene combination of *DXS*+*HDR*+*GGPPS* (Figure 3E), consistent with the hypothesis that OH-GGPP is produced when HMBPP reduction is limiting due to lack of HDR activity.

### Transient Expression in *N. benthamiana* of a *GERANYLLINALOOL SYNTHASE* From *Nicotiana attenuata* (*NaGLS*) Results in Production of Both Geranyllinalool and 16-Hydroxy-Geranyllinalool When Co-expressed With *DXS*+*GGPPS* But Only Geranyllinalool When *HDR* Is Included in the Gene Combination

**Figure 4:**
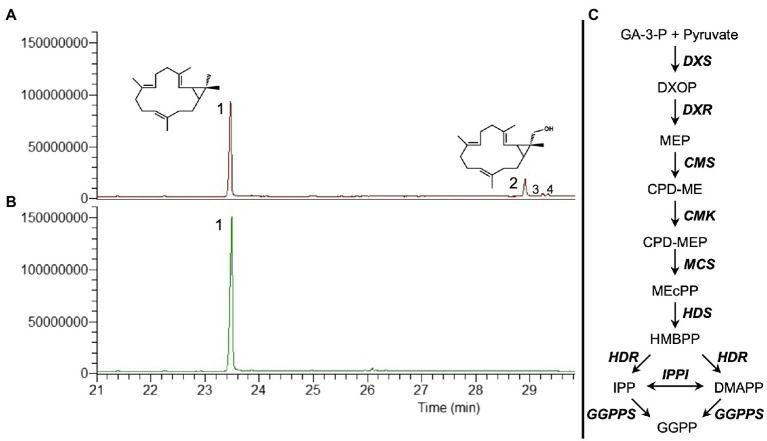
*NaGLS* produces both geranyllinalool and 16-hydroxy-geranyllinalool in *Nicotiana benthamiana* **(A)** GC–MS TIC of extract from *N. benthamiana* infiltrated with *DXS*+*GGPPS*+*NaGLS*. **(B)** Extract from material infiltrated with *DXS*+*HDR*+*GGPPS*+*NaGLS*. **(C)** Quantities of compounds 1, 2, and 3 in extracts from the different gene combinations (ng/mg DW±SD, *n*=5). Symbols show significant differences between treatment means (*p*<0.05, *F*-test and *T*-test). 1, geranyllinalool; 2, 16-hydroxy-geranyllinalool; and 3, undetermined.

To further explore whether 16-OH-GGPP could be used by other diterpene synthases, we transiently expressed the *Nicotiana attenuata* geranyllinalool synthase (*NaGLS*) in *N. benthamiana*, alone or in combination with *DXS*+*HDR*+*GGPPS* (Figure 4 and Supplementary Figure S3). This resulted in accumulation of geranyllinalool in both cases. Co-expression of *NaGLS*+*DXS*+*GGPPS* produced geranyllinalool but also two additional peaks with retention times (R*_t_*) of 30.0 and 31.6min (Figure 4A and Supplementary Figure S3E). We used UPLC-MS (Supplementary Figure S4) and flash chromatography to purify the compound giving rise to the larger peak at Rt 30.0 min in sufficient quantities to permit its identification by NMR spectroscopy as 16-OH-geranyllinalool (Supplementary Figure S5) This novel natural product represented up to 25% of the geranyllinalool peak (Figure 4C). This is comparable amount of 16-OH-casbene to casbene (30%) with the same reduced gene combination (Supplementary Figure S2).

Despite obvious parallels with the regio-isomeric 17-OH-geranyllinalool (Supplementary Figure S6), the precursor of the insecticidal diterpene glycosides in many *Nicotiana* species (Snook et al., 1997; Jassbi et al., 2010; Falara et al., 2014), there is no indication that 16-OH-geranyllinalool could be involved in the biosynthetic pathway to 17-OH-diterpenes. A recent work actually demonstrated that two cytochrome P450s from *N. attenuata* are responsible for the 17-hydroxylation of geranyllinalool (Li et al., 2021).

We did not detect any other hydroxy geranyllinalool compounds apart from 16-OH-geranyllinalool with the *DXS*+*GGPPS*+*NaGLS* gene combination, providing further evidence that 16-OH-geranyllinalool is derived from a direct conversion of 16-OH-GGPP.

## Discussion

This work provides evidence for the formation of 16-OH-GGPP when the flux through the MEP pathway in *N. benthamiana* is altered. Both casbene synthase from *Jatropha curcas* and geranyllinalool synthase from *N. attenuata* result in production of 16-hydroxylated versions of their usual diterpene products when transiently expressed in *N. benthamiana* producing 16-OH-GGPP. The detection of additional minor compounds from both enzymes suggests that additional products may also arise when 16-OH-GGPP is used as substrate. We hypothesize that 16-OH-GGPP is formed through the action of *A. thaliana* GGPP synthase when HMBPP levels are elevated due to increased flux through the MEP pathway and a bottleneck exists at the HDR step. When the HDR enzyme, which reduces the hydroxy group in HMBPP to make DMAPP, is co-expressed with DXS and GGPPS, neither 16-OH-GGPP nor 16-OH-diterpenes are detected. Taken together the evidence presented supports formation of 16-OH-casbene or 16-OH-geranyllinalool by promiscuous diterpene synthases acting on 16-OH-GGPP rather than P450-based hydroxylation of casbene or geranyllinalool. The fact that formation of these 16-hydroxylated compounds is exclusively associated with over-expression of the first step in the MEP pathway combined with omission of the last step points to the bottleneck at the HDR step giving rise to 16-OH-GGPP *via* a plausible route. Interestingly, in *Escherichia coli*, overproduction of HMBPP is cytotoxic and removal of this effect is achieved by activation of *IspG*, the gene encoding the *HDR* equivalent in plants (Li et al., 2017). The transient expression approach we use *in planta* may have by-passed such regulation if indeed it is important in *N. benthamiana*.

There are precedents from the terpenoid literature for the formation of more highly oxidized precursors, which are then accepted as alternatives to the normal substrate in a known biosynthetic pathway. Thus, 2,3-oxidosqualene, the usual precursor of triterpenes and sterols, can undergo a second oxidization by the endogenous squalene epoxidase to form dioxidosqualene, when it accumulates in yeast (Salmon et al., 2016). Research has shown that a mutated triterpene synthase actually prefers this double-oxygenated substrate to the normal 2,3-oxidosqualene, leading to the production of unusual triterpenes that incorporate an additional oxygen atom in the fifth ring (Salmon et al., 2016). There are also examples of synthetic chemistry work focusing on obtaining analogues of the sesquiterpene precursor farnesyl pyrophosphate (Dolence and Dale Poulter, 1996; Placzek and Gibbs, 2011) or even (Z,E,E)-geranylgeranyl pyrophosphate (Minutolo et al., 2006), demonstrating the interest of alternative substrates for terpenoid production.

It is perhaps unlikely that 16-OH-GGPP is a significant substrate in nature when the MEP and diterpene biosynthetic pathways are subject to their normal mechanisms of regulation. However, the substantial level of 16-hydroxylated diterpenes with native diterpenes synthases in transient expression systems might suggest that 16-OH-diterpenes can become more biologically relevant under abnormal circumstances, when such regulation is compromised.

In terms of engineering biology, this work demonstrates the importance of regulating flux through biosynthetic pathways to ensure intermediates do not accumulate as the promiscuity of substrate specificity can result in the production of unexpected end products. On the other hand, this example shows that the generation of a novel GGPP substrate can open the possibility of entirely new diterpenes that could be further modified and evaluated in terms of their bioactivity.

## Data Availability Statement

The original contributions presented in the study are included in the article/Supplementary Material; further inquiries can be directed to the corresponding author.

## Author Contributions

EF designed experiments, performed experiments, and analyzed data. GB, DH, and TL performed experiments, analyzed data, and contributed to the writing of the manuscript. EF and IG wrote the manuscript. IG contributed to the design and analysis of the study. All authors contributed to the article and approved the submitted version.

## Funding

This research was funded by the BBSRC and Innovate UK under grant number BB/M018210/01. High-resolution mass spectrometry was performed using equipment within the Centre of Excellence in Mass Spectrometry (University of York), funded by Science City York (Yorkshire Forward, EP/K039660/1, EP/M028127/1).

## Conflict of Interest

The authors declare that the research was conducted in the absence of any commercial or financial relationships that could be construed as a potential conflict of interest.

## Publisher’s Note

All claims expressed in this article are solely those of the authors and do not necessarily represent those of their affiliated organizations, or those of the publisher, the editors and the reviewers. Any product that may be evaluated in this article, or claim that may be made by its manufacturer, is not guaranteed or endorsed by the publisher.
