# Supplementary material for: Engineering Production of a Novel Diterpene Synthase Precursor in Nicotiana benthamiana
Source: Front Plant Sci. 2021 Oct 20;12:757186. doi: 10.3389/fpls.2021.757186 (PMC8564105; doi:10.3389/fpls.2021.757186)
Supplement: Supplementary file 1 [file Data_Sheet_1.docx]

**Figure S1 NMR data for 16-OH-casbene.**

Data for 16-Hydroxy-casbene:^1^H NMR (700 MHz, CDCl_3_): δ 4.98 (t, J = 7 Hz, 1H (H-11)), 4.89 (t, J = 7 Hz, 1H (H-7)), 4.88 (d, J = 8 Hz, 1H (H-3)), 3.49 (br, 1H (16-OH)), 3.38 (d, J = 11 Hz, 1H (H-16)), 3.35 (d, J = 11 Hz, 1H (H-16)), 2.28 (m, 1H (H-6)), 2.21 (m, 1H (H-5)), 2.19 (m, 1H (H-13)), 2.12 (m, 2H (H-9 and H-10)), 2.10 (m, 1H, (H-5)), 2.08 (m, 1H (H-6)), 2.04 (m, 1H (H-10)), 1.91 (m, 1H (H-9)), 1.88 (m, 1H (H-13)), 1.75 (m, 1H (H-14)), 1.66 (s, 3H (H-18)), 1.58 (s, 3H (H-20)), 1.56 (s, 3H (H-19)), 1.37 (dd, J = 9, 8, Hz, 1H (H-2)), 1.05 (s, 3H (H-17)), 1.01 (m, 1H (H-14)), 0.72 (ddd, J = 11, 9, 2 Hz, 1H (H-1));^13^C NMR (175 MHz, CDCl_3_): δ 137.2 (C-4), 135.2 (C-12), 133.5 (C-8), 125.4 (C-7), 123.6 (C-11), 119.7 (C-3), 73.5 (C-16), 40.1 (C-13), 39.5 (C-9), 39.3 (C-5), 27.1 (C-1), 26.6 (C-15), 24.9 (C-6), 24.0 (C-10), 23.7 (C-14), 22.7 (C-2), 16.5 (C-20), 16.3 (C-18), 15.7 (C-19), 11.3 (C-17).

^1^H NMR (700 MHz)


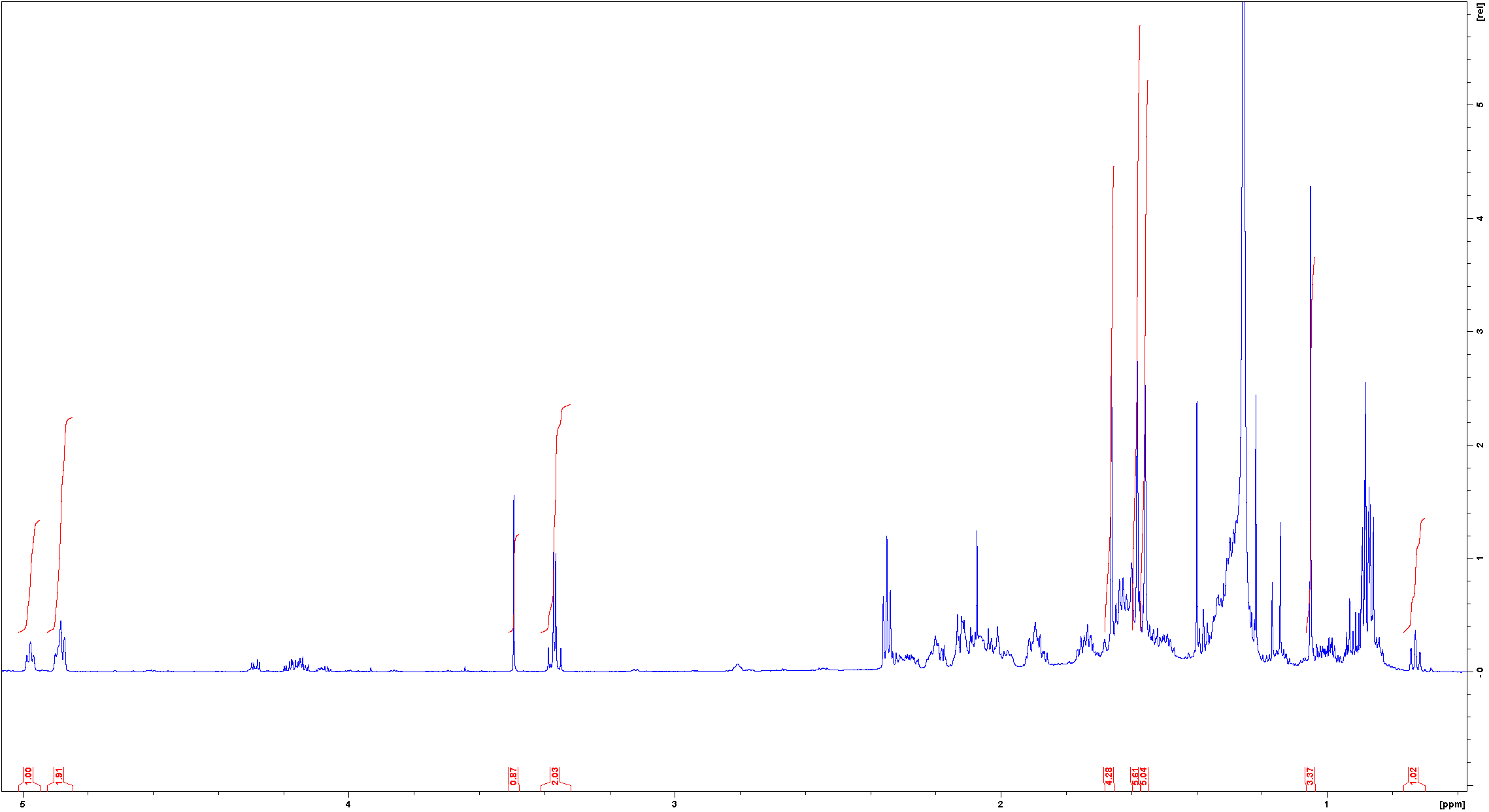


^13^C NMR (175 MHz)


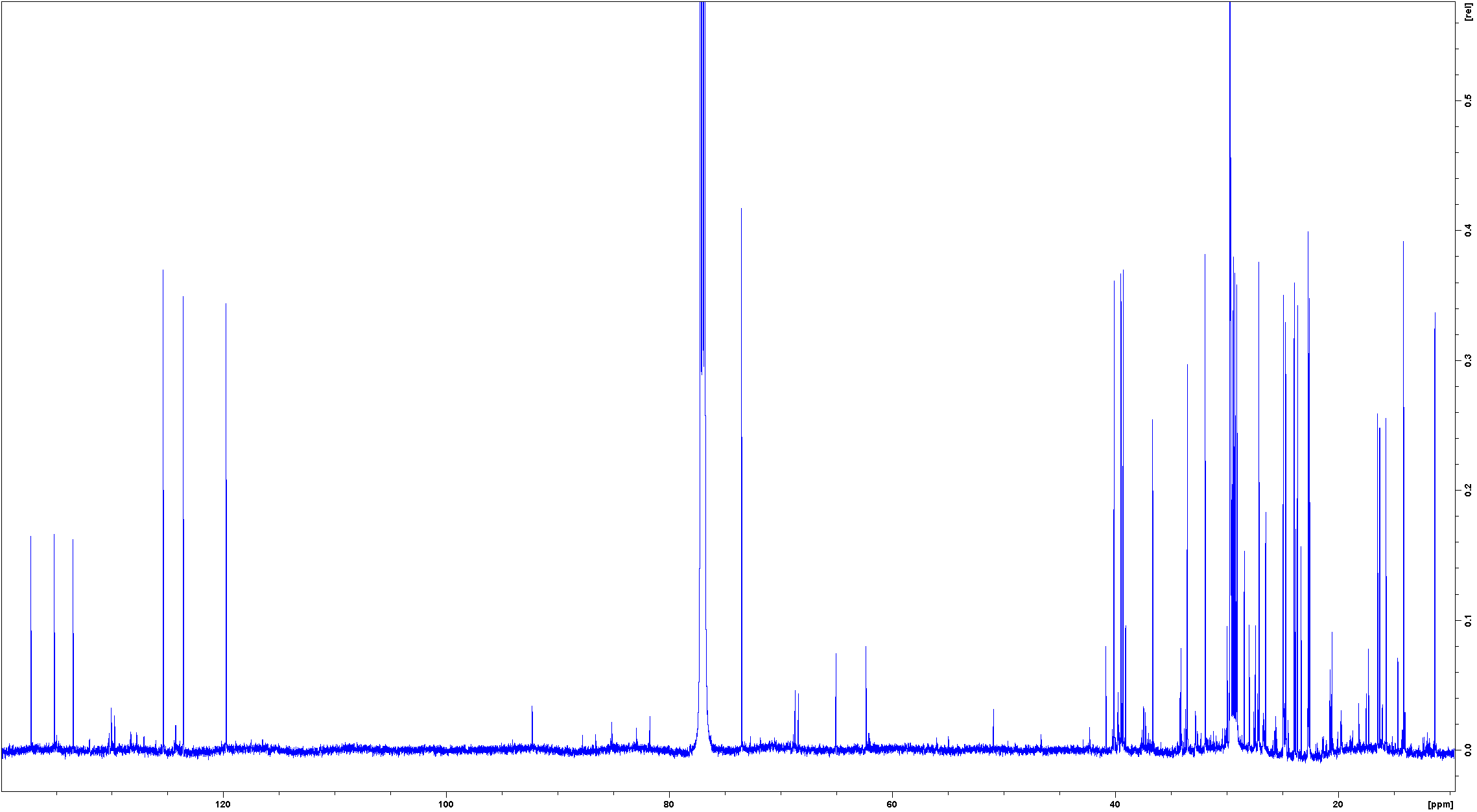


Edited-HSQC


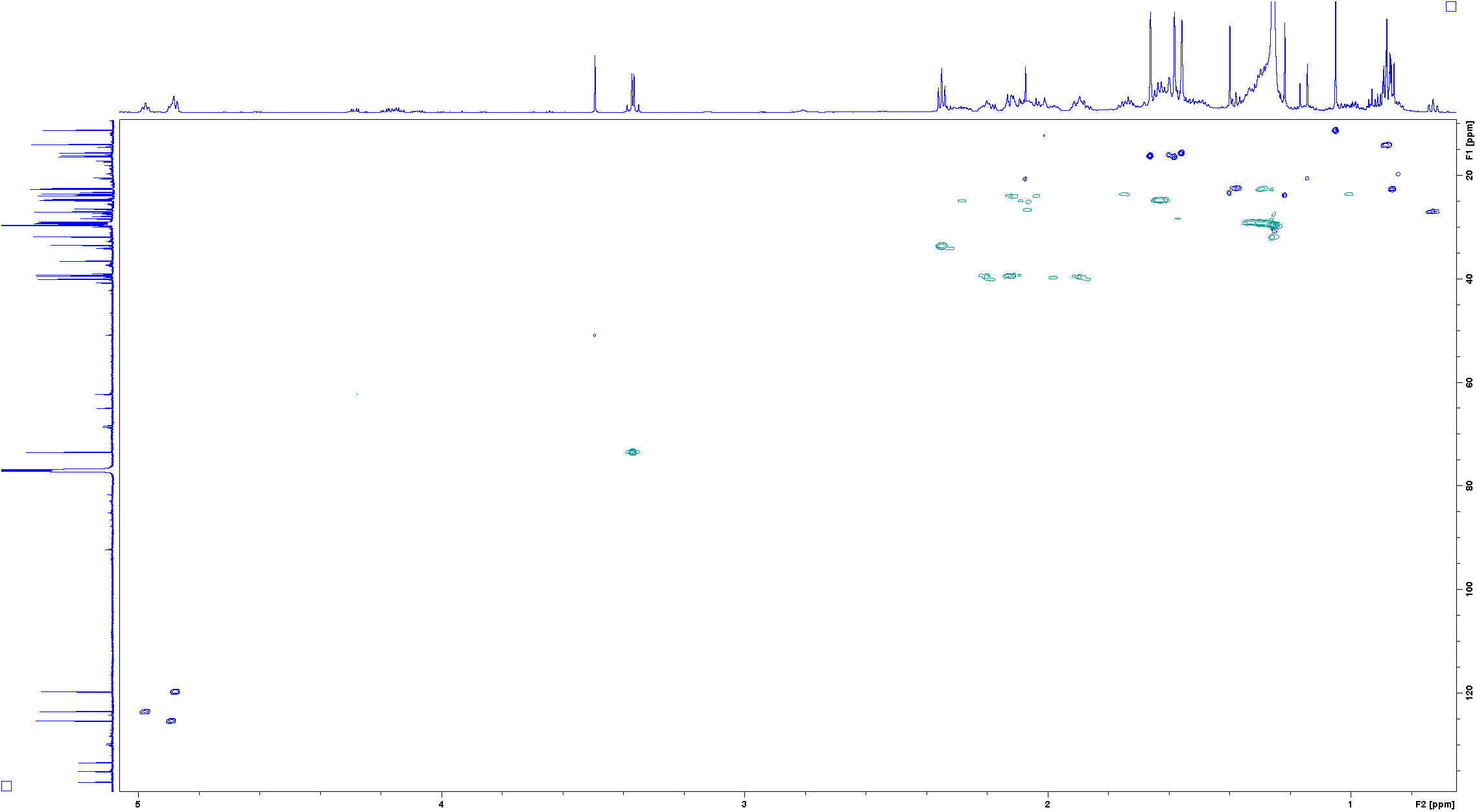


HMBC


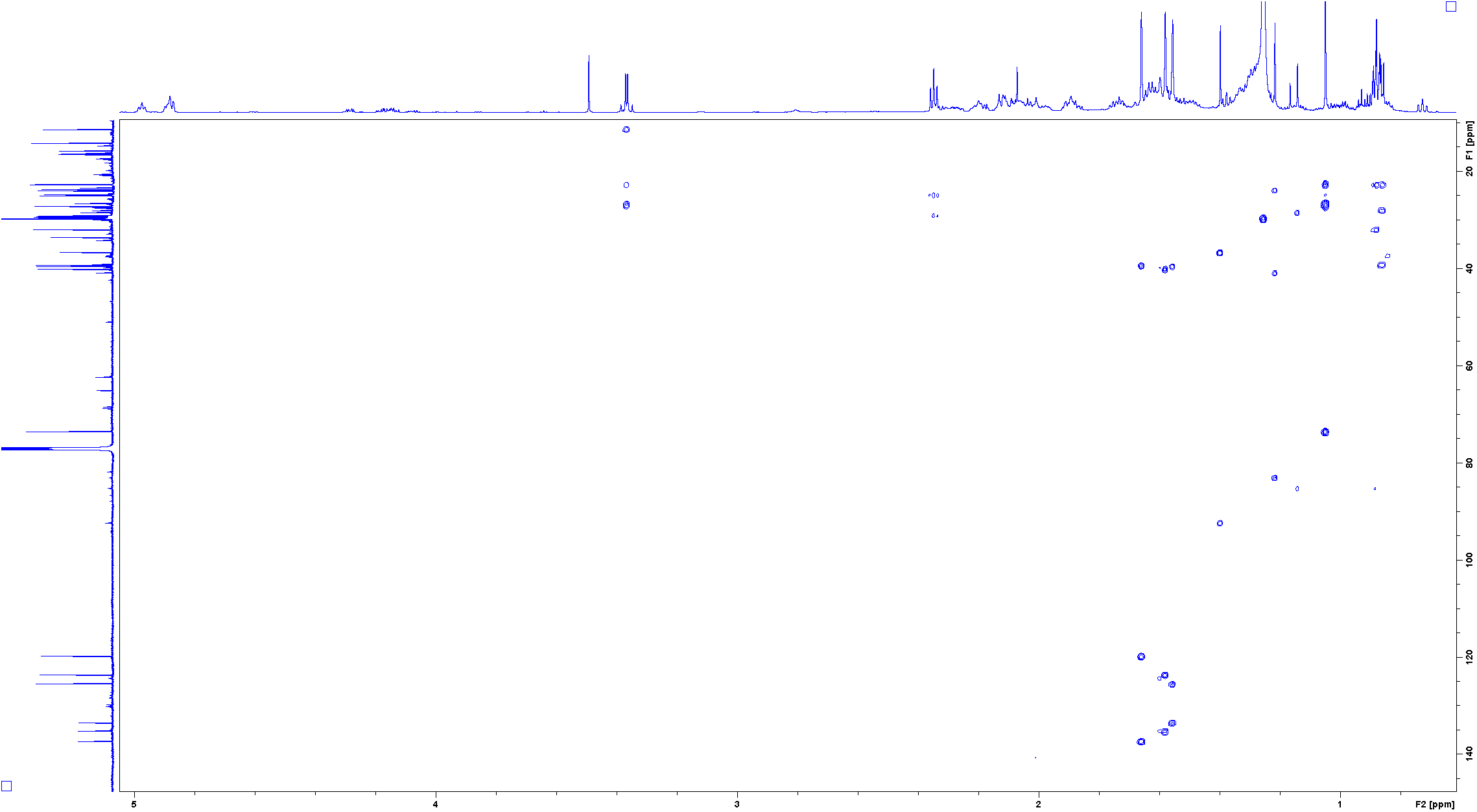


COSY


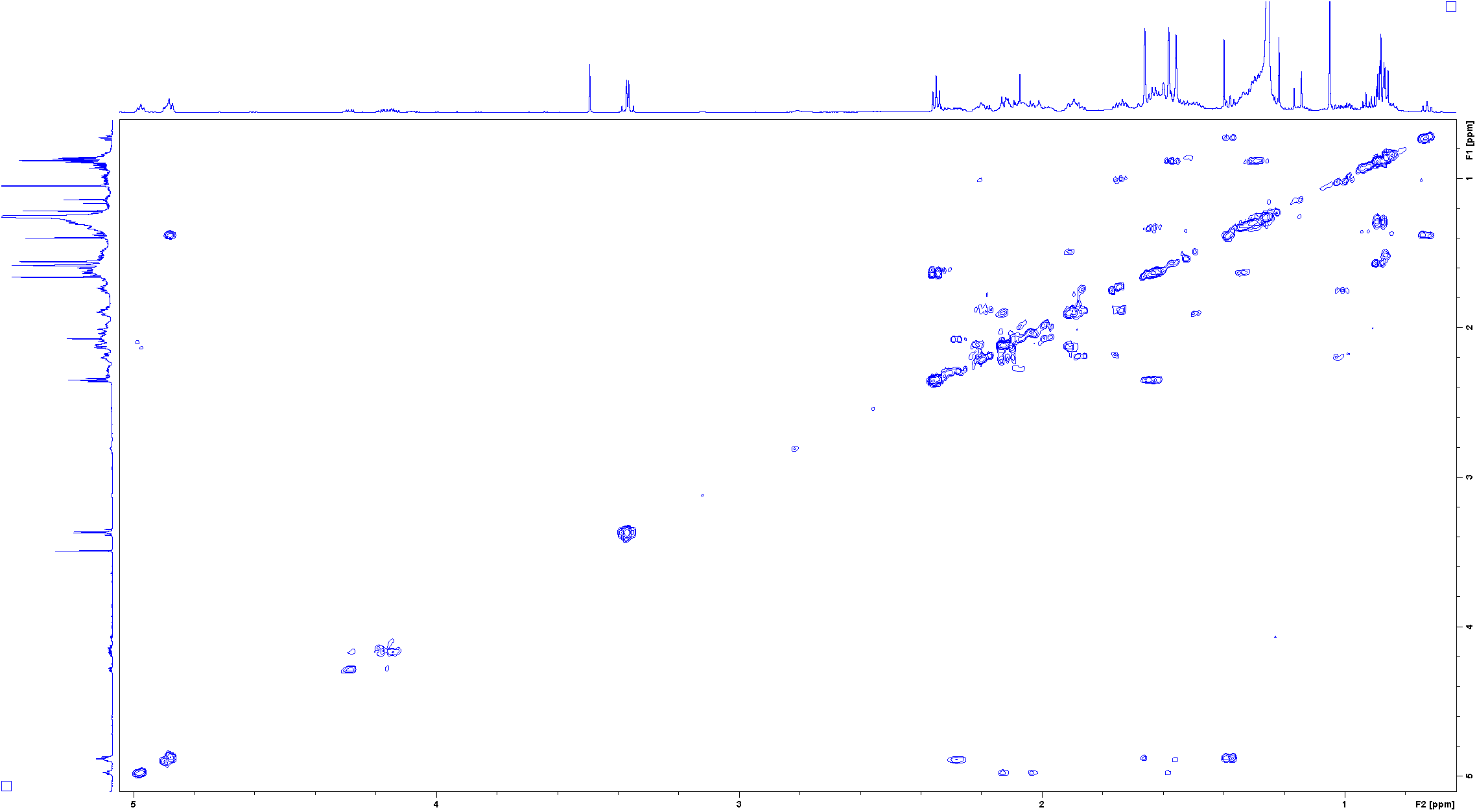


NOESY


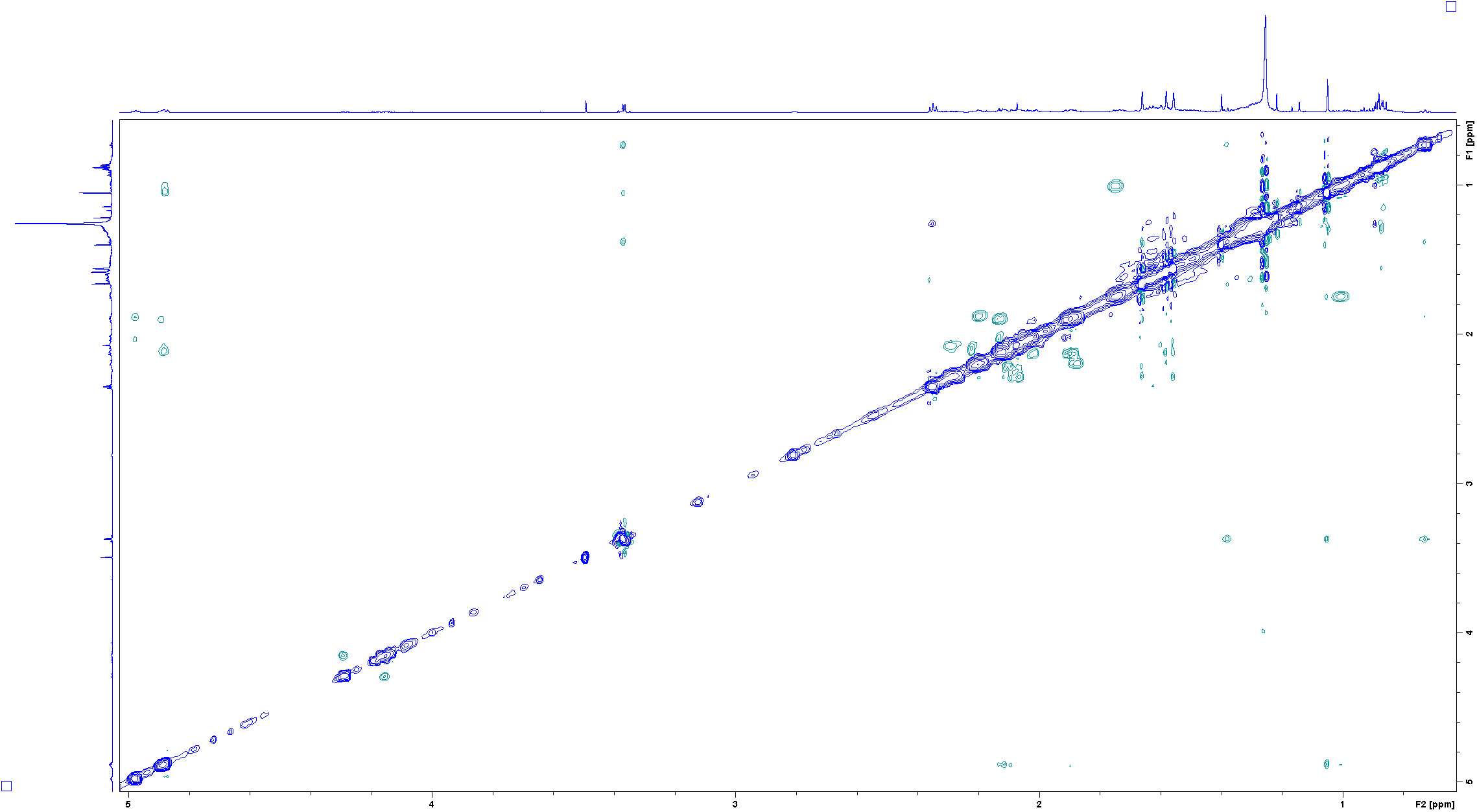


**Figure S2 Amounts of casbene and 16-OH-casbene produced following co-infiltration of different combinations of diterpenoid precursor genes with casbene synthase.** Co-infiltration of *AtDXS* + *AtGGPPS* + *JcCAS* compared to *AtDXS* + *AtHDR* + *AtGGPPS* + *JcCAS*. Average content (in ng/mg DW) ± standard deviations are displayed (n = 3). Symbols show significant differences between treatment means (P < 0.05, F-test and T-test).


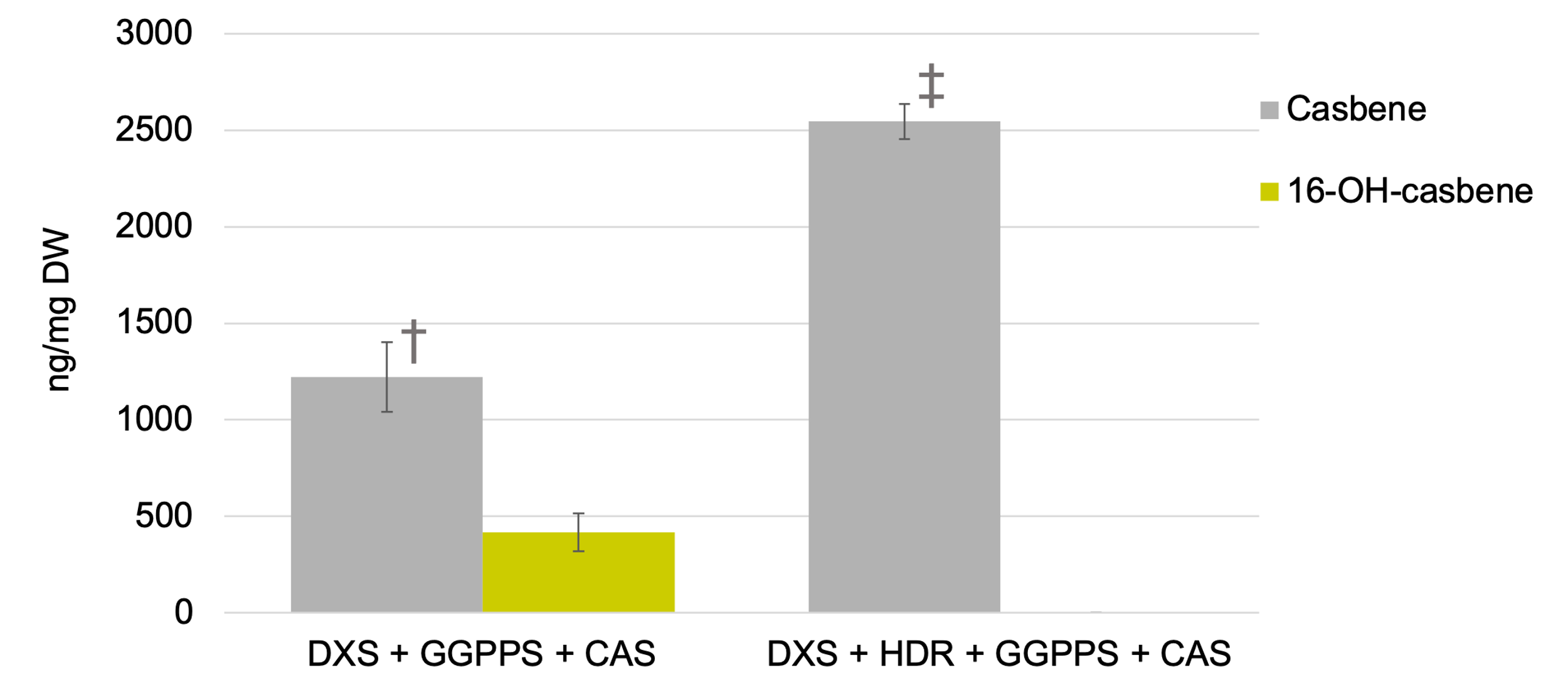


**Figure S3 GC-MS TIC of extracts from *N. benthamiana* infiltrated with different gene combinations** (A) Ethyl acetate extract of *N. benthamiana* infiltrated with empty vector control. (B) Infiltration with *DXS* + *GGPPS*. (C) Infiltration with *DXS* + *HDR* + *GGPPS*. (D) Infiltration with *NaGLS*. (E) Infiltration with DXS + *GGPPS* + *NaGLS*. (F) Infiltration with *DXS* + *HDR* + *GGPPS* + *NaGLS*. (G) Authentic standard of geranyllinalool.

1, geranyllinalool; 2, 16-hydroxy-geranyllinalool; 3, unknown compound.

(A)


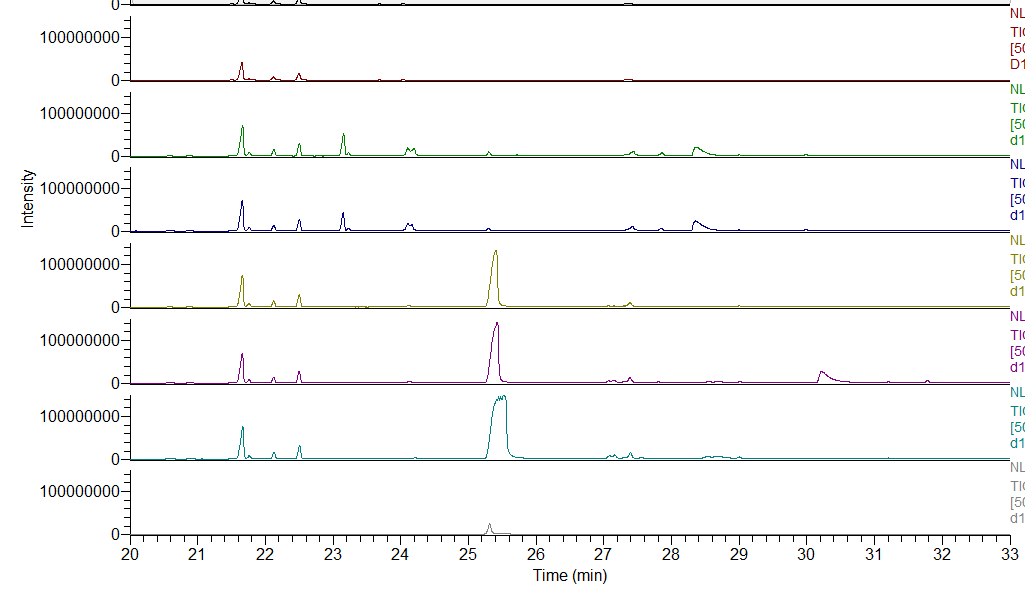


(B)

(C)

(D)

1

(E)

1

3

2

(F)

1

(G)

1

**Figure S4 UPLC-MS TIC of extracts from *N. benthamiana* infiltrated with different gene combinations and mass spectra of geranyllinalool and 16-hydroxyl-geranyllinalool.** (A) Ethyl acetate extract of *N. benthamiana* infiltrated with empty vector control. (B) Infiltration with *DXS* + *GGPPS*. (C) Infiltration with *DXS* + *HDR* + *GGPPS*. (D) Infiltration with *NaGLS*. (E) Infiltration with *DXS* + *GGPPS* + *NaGLS*. (F) Infiltration with *DXS* + *HDR* + *GGPPS* + *NaGLS*. (G) Authentic standard of geranyllinalool. (H) Mass spectrum of geranyllinalool (1) and 16-hydroxy-geranyllinalool (2).


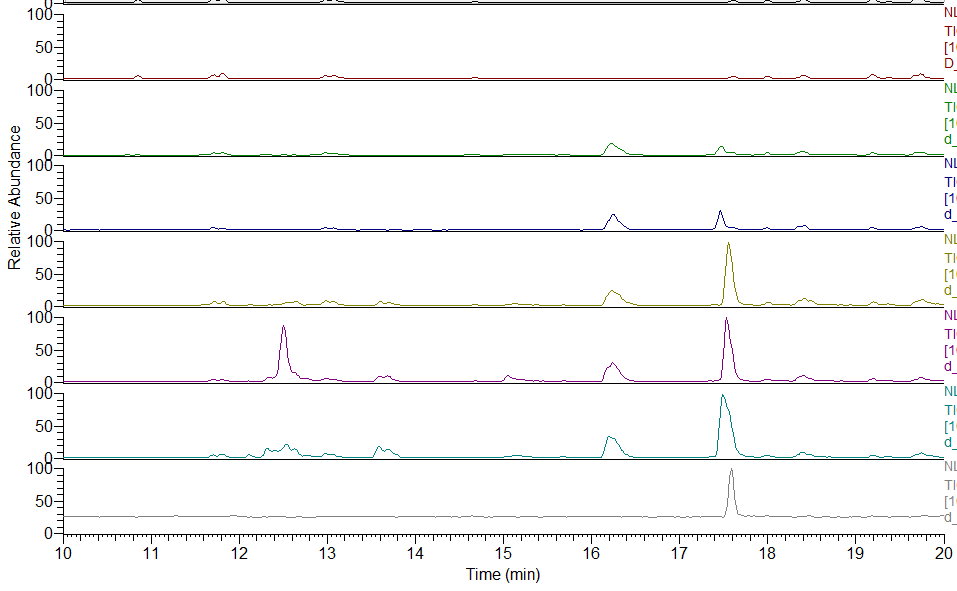


(A)

(B)

(C)


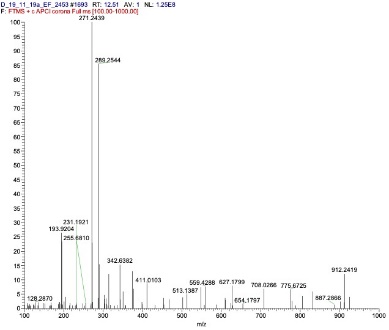

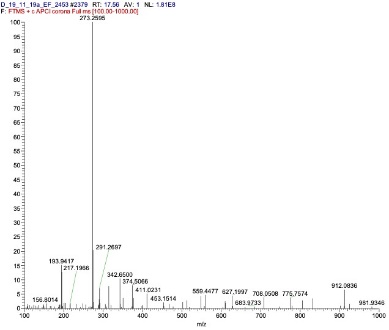


(H)

(D)

(E)

(F)

(G)

1

1

1

1

2

2

1

**Figure S5 NMR data for 16-hydroxy-geranyllinalool.**

Data for 16-hydroxy-granyllinalool: ^1^H NMR (700 MHz, CDCl_3_): δ 5.92 (dd, J = 17, 11 Hz, 1H (H-2)), 5.39 (ttq, J = 7, 1, 1 Hz, 1H (H-14)), 5.21 (dd, J = 17, 1 Hz, 1H (H-1)), 5.14 (ttq, J = 7, 1, 1 Hz, 1H (H-6)), 5.10 (ttq, J = 7, 1, 1 Hz, 1H (H-10)), 5.06 (dd, J = 11, 1 Hz, 1H (H-1)), 3.99 (br s, 2H (H-16)), 2.13 (dt, J = 7, 7 Hz, 2H (H-13)), 2.07 (m, 4H (H-5 and H-9)), 2.01 (m, 2H (H-12)), 1.99 (m, 2H (H-8)),1.67 (br s, 3H (H-17)), 1.603 (br s, 3H (H-19)), 1.600 (br s, 3H (H-18)), 1.58 (m, 2H (H-4)), 1.28 (s, 3H (H-20));^13^C NMR (175 MHz, CDCl_3_): δ 145.0 (C-2), 135.5 (C-7), 134.7 (C-15), 134.6 (C-11), 126.1 (C-14), 124.4 (C-10), 124.3 (C-6), 111.7 (C-1), 73.5 (C-3), 69.0 (C-16), 42.0 (C-4), 39.6 (C-8), 39.3 (C-12), 27.9 (C-20), 26.5 (C-9), 26.2 (C-13), 22.7 (C-5), 16.03 (C-18), 16.00 (C-19), 13.7 (C-17).

^1^H NMR (500 MHz)


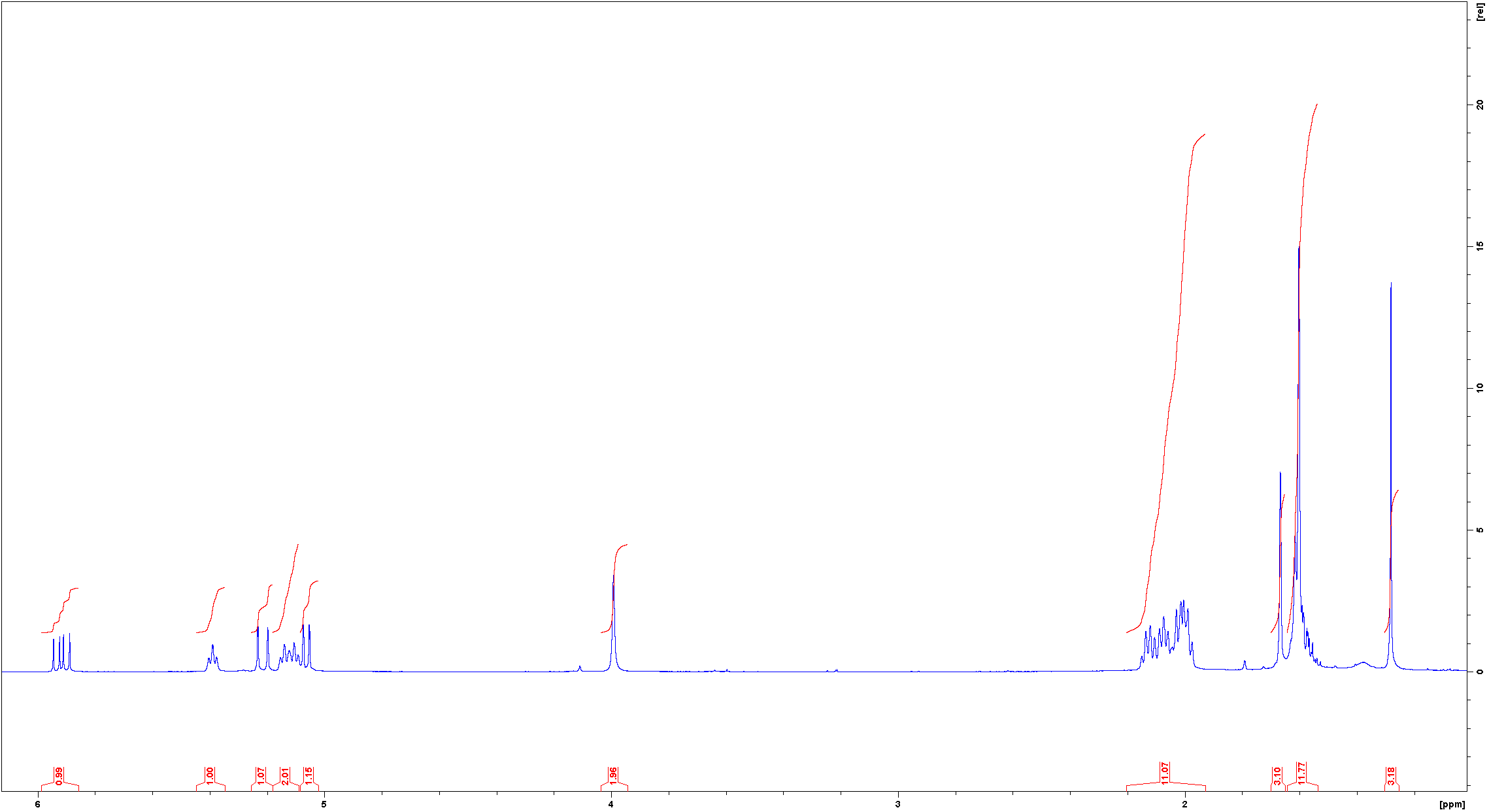


^13^C NMR (125 MHz)


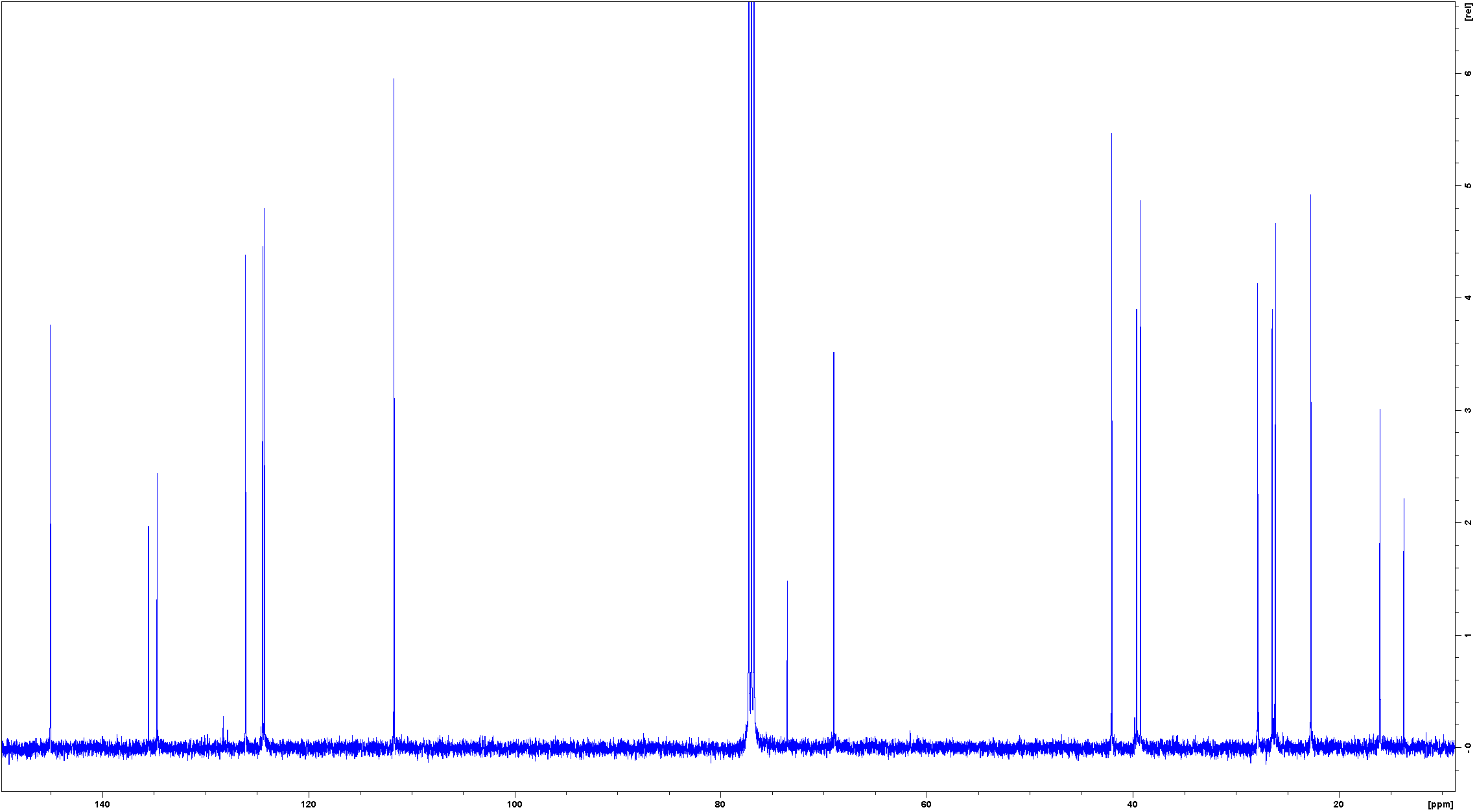


Edited-HSQC


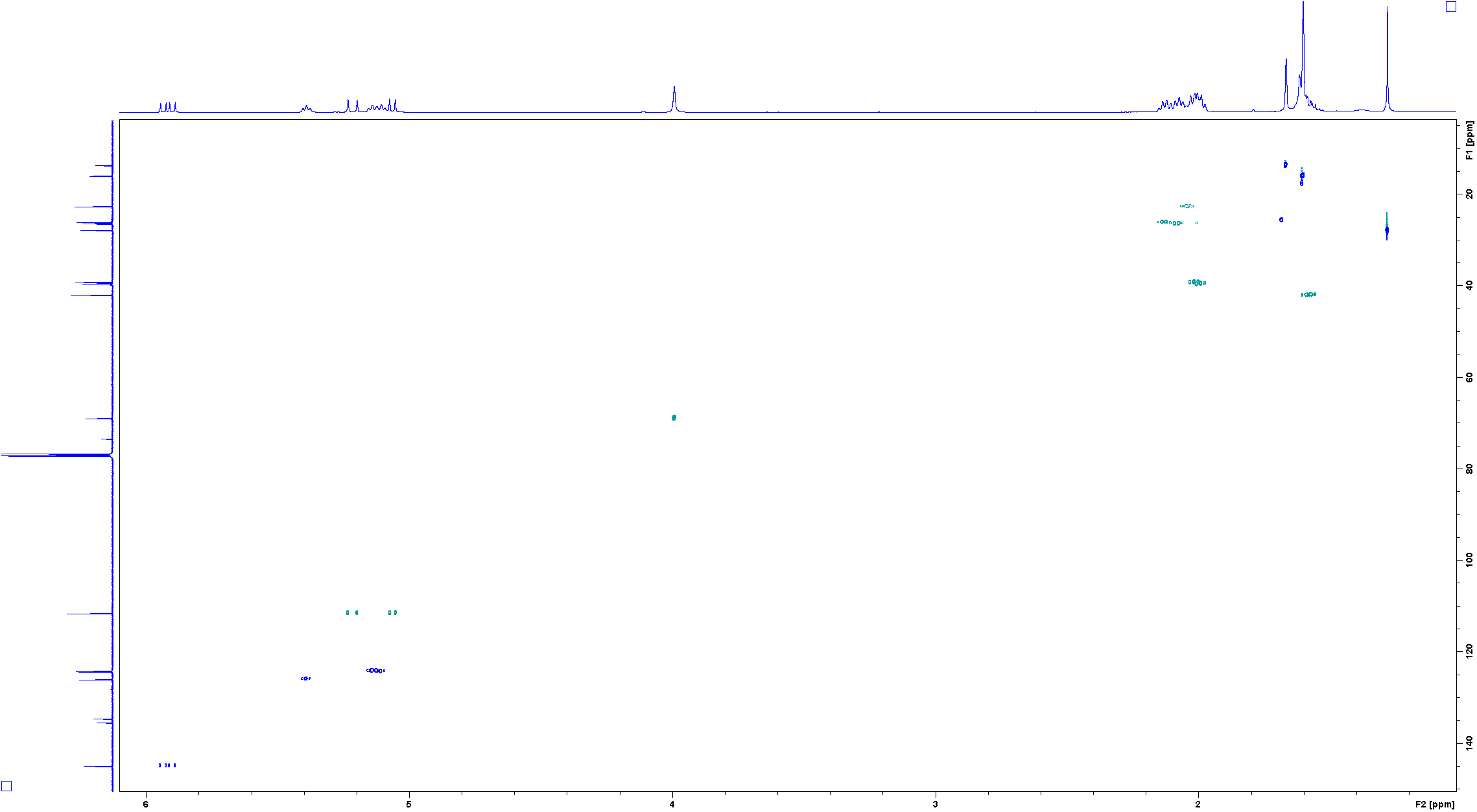


HMBC


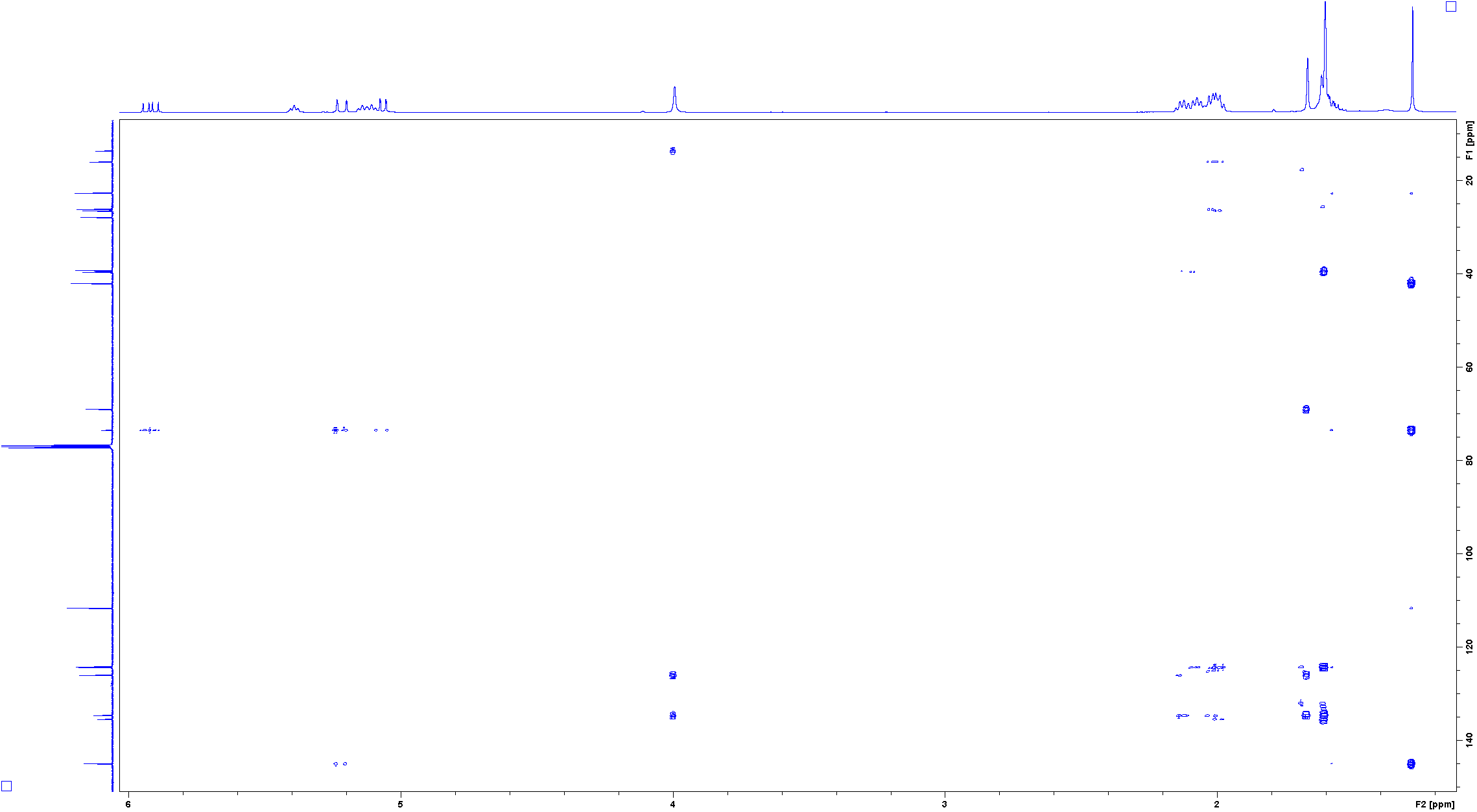


COSY


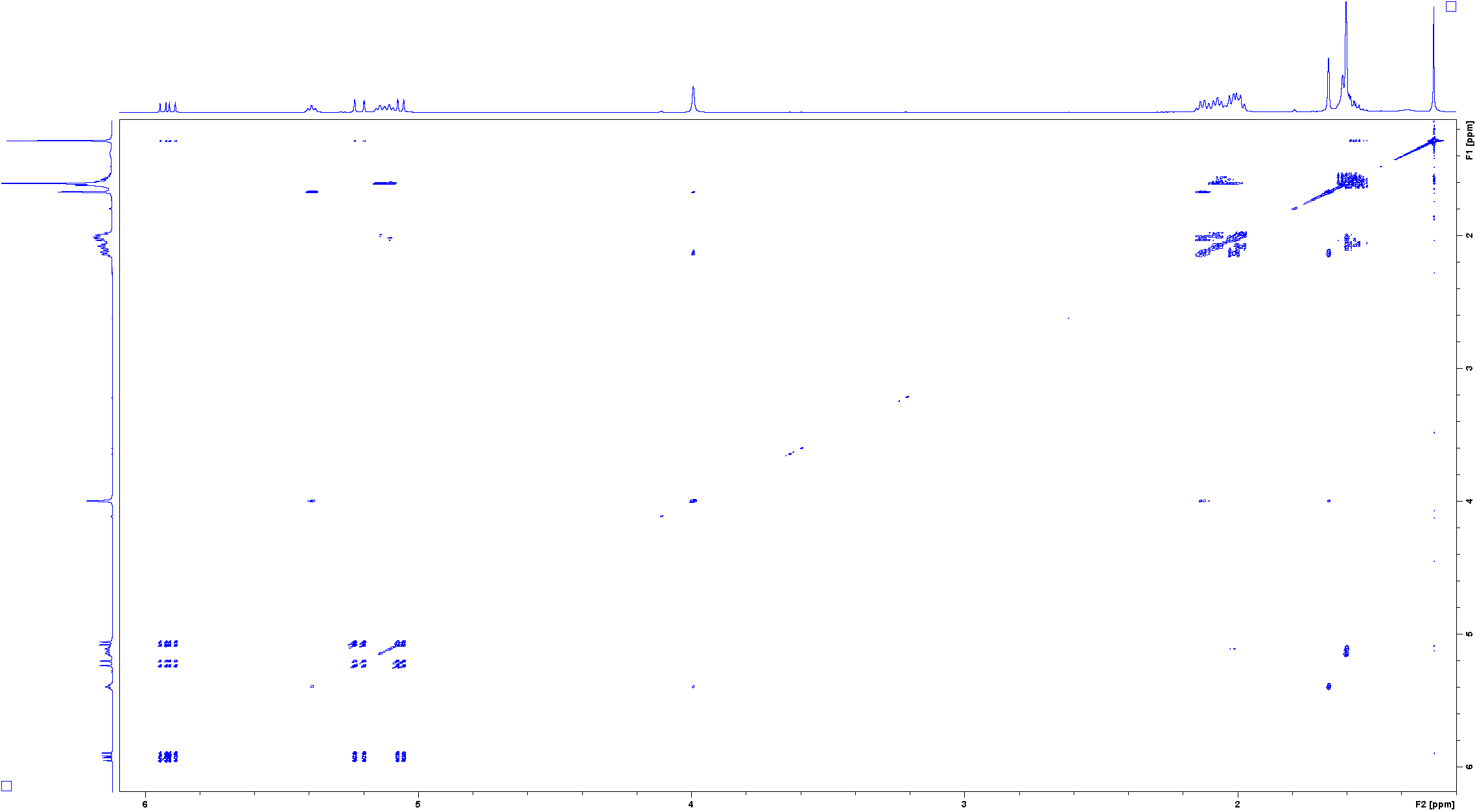


NOESY


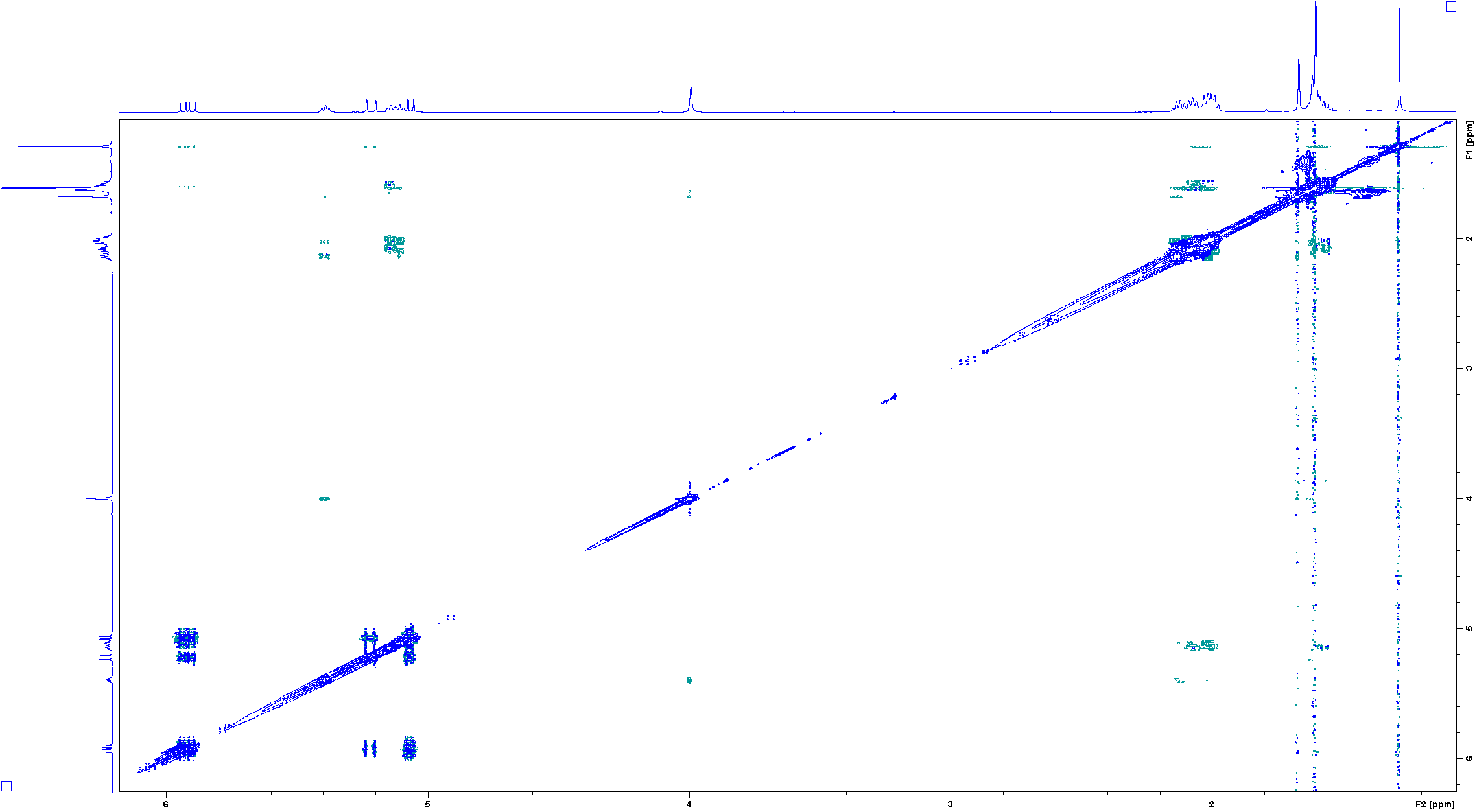


**Figure S6 Structure of 16-hydroxy-geranyllinalool (A) and 17-hydroxy-geranyllinalool (B).**


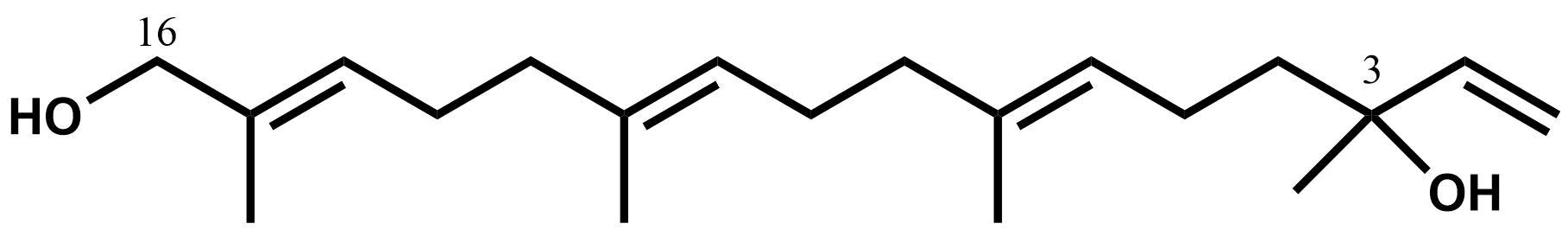


(A)


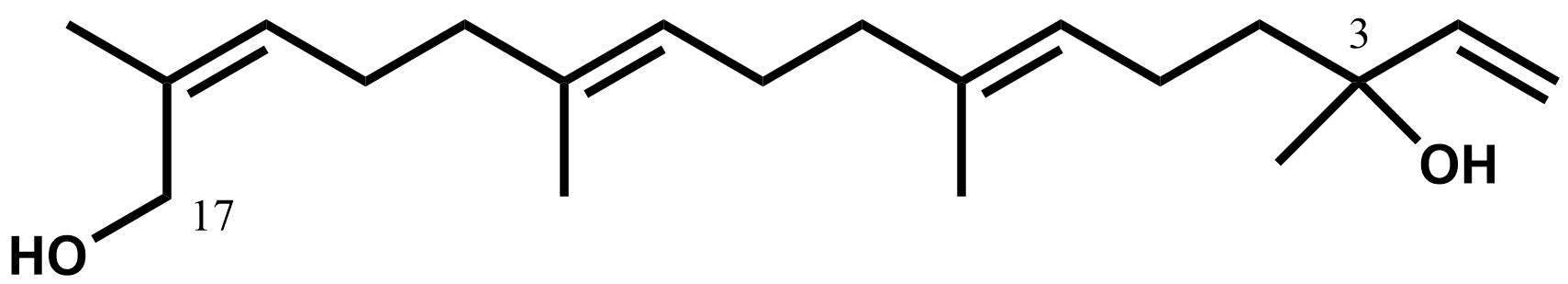


(B)
